# The Role of Anaphase-Promoting Complex/Cyclosome (APC/C) in Plant Reproduction

**DOI:** 10.3389/fpls.2021.642934

**Published:** 2021-02-24

**Authors:** Marina de Lyra Soriano Saleme, Ingrid Rocha Andrade, Nubia Barbosa Eloy

**Affiliations:** Department of Biological Sciences, Escola Superior de Agricultura “Luiz de Queiroz,” University of São Paulo, Piracicaba, Brazil

**Keywords:** plant development, cell cycle, anaphase-promoting complex/cyclosome, gametogenesis, sexual reproduction

## Abstract

Most eukaryotic species propagate through sexual reproduction that requires male and female gametes. In flowering plants, it starts through a single round of DNA replication (S phase) and two consecutive chromosome segregation (meiosis I and II). Subsequently, haploid mitotic divisions occur, which results in a male gametophyte (pollen grain) and a female gametophyte (embryo sac) formation. In order to obtain viable gametophytes, accurate chromosome segregation is crucial to ensure ploidy stability. A precise gametogenesis progression is tightly regulated in plants and is controlled by multiple mechanisms to guarantee a correct evolution through meiotic cell division and sexual differentiation. In the past years, research in the field has shown an important role of the conserved E3-ubiquitin ligase complex, Anaphase-Promoting Complex/Cyclosome (APC/C), in this process. The APC/C is a multi-subunit complex that targets proteins for degradation via proteasome 26S. The functional characterization of APC/C subunits in Arabidopsis, which is one of the main E3 ubiquitin ligase that controls cell cycle, has revealed that all subunits investigated so far are essential for gametophytic development and/or embryogenesis.

## Introduction

A crucial stage in the life cycle of living organisms, which include angiosperms (flowering plants), is the capacity of ensuring species continuation through a reproductive system. Sexual reproduction is one of the main processes that leads to self-perpetuation of species. Moreover, it provides genetic diversity over the progeny, which represents the basis for plant adaptation in different environments (Schmidt et al., 2015; Sprunck, 2020). The keystone of this process is the formation of gametes (male and female), called haploid cells, that fuse to form a diploid somatic cell, the zygote (Dresselhaus et al., 2016).

**FIGURE 1:**
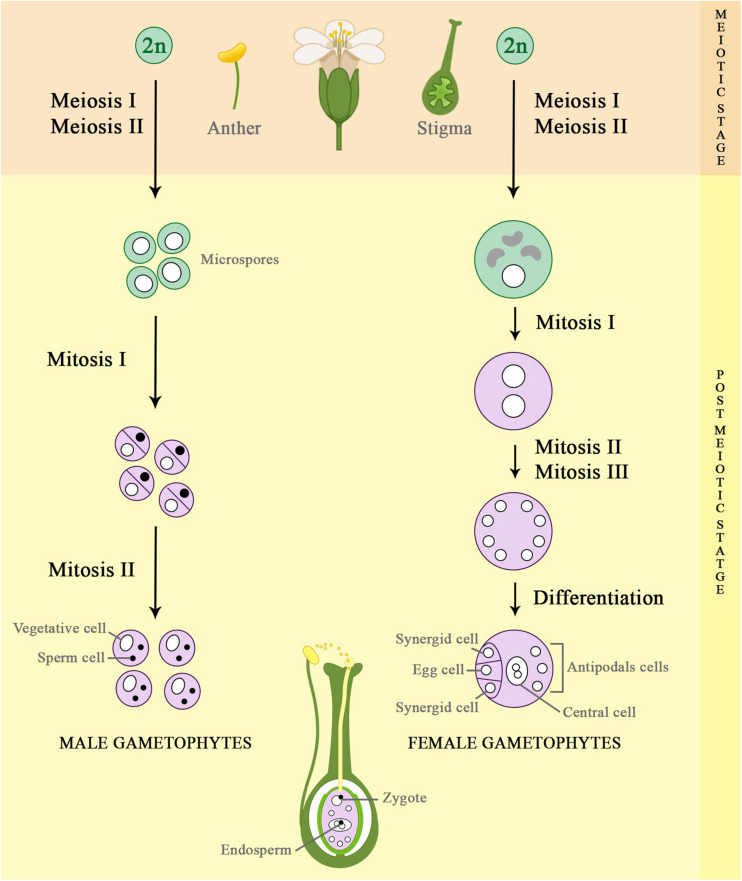
Schematic representation of female and male gametogenesis process. The propagation of flowering plants through sexual reproduction is an essential process during plant development, which results in genetic variability among the offspring. The gamete formation process starts with diploid cells (2n), which undergo meiosis I and II, dividing their number of chromosomes by half (n). Then, the haploid cells formed will divide and differentiate by multiple mitotic cell divisions, forming the male and female gametophytes (pollen grains and embryo sac, respectively) that further will generates the zygote and the endosperm, which characterize the double fertilization in plants.

The formation of gametes starts with a singular process known as meiosis (Figure 1), characterized by a reduction in the ploidy level of the original cell by halving the nuclear DNA content in two subsequent chromosome segregation steps, without an interfering S phase (Wijnker and Schnittger, 2013). In plants, meiosis occurs only during sexual reproduction in specialized cells. It gives rise to four haploid gametes, which are different from the original ones, because the process allows for exchanges of genetic material by recombination (Mercier et al., 2015; Wang and Copenhaver, 2018). The gametes are formed during floral organ development from somatic tissues of adult plants (sporophyte). The sporophyte mother cells differentiate within the anther (pollen mother cell) and the ovule (megaspore mother cell) to form male (pollen grains) and female (embryo sac) gametophytes, respectively (Ma and Sundaresan, 2010; Berger and Twell, 2011).

**FIGURE 2:**
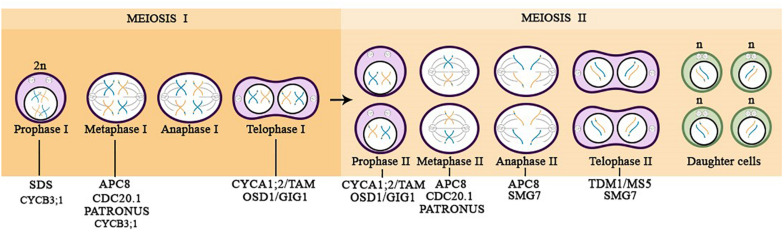
Overview of the meiotic progression in diploid plants highlighting the APC/C subunit (APC8), regulators (CDC20.1 and SAMBA), and substrates acting during the specific phases of the meiotic cell division. The illustration is based on researches carried out in the model plant *Arabidopsis thaliana*.

In summary, gametophyte development comprises two main stages (Figure 1), known as meiotic and post-meiotic (Ma and Sundaresan, 2010; Berger and Twell, 2011). The meiotic stage consists of only one round of DNA replication (S phase) and two successive divisions (meiosis I and meiosis II). During the first meiotic division, homologous chromosomes are separated, whereas in meiosis II sister chromatids segregate to opposite poles, resulting in four haploid daughter cells (gametes), in case of diploid plants (Figure 2). Therefore, each gamete presents half of the amount of genomic DNA of a sporophyte, which is important to avoid genome duplication in every new generation (Zamariola et al., 2014; Mercier et al., 2015).

After meiosis, the fate of male and female meiotic products is different (Figure 1). During female gametophyte development, usually only one out of four haploid daughter cells formed gives rise to a functional megaspore mother cell that undergoes three rounds of mitotic divisions, generating an eight-nucleate embryo sac. Afterward, the embryo sac goes through cellularization and cell specification, producing seven cells, which belong to four cell types: two gametic cells known as egg cell (1n) and central cell (2n, homodiploid); and two accessory cell types, which are two synergids and three antipodals (Yang et al., 2010; Sprunck and Groß-Hardt, 2011; Skinner and Sundaresan, 2018). However, for male gametogenesis, each of the four meiotic products (microspore) undergoes mitotic division to produce a generative cell and a vegetative cell. The generative cell undergoes another mitotic division, giving rise to twin sperm cells, whereas the vegetative cell does not divide further (McCormick, 2004; Scott et al., 2004; Berger and Twell, 2011; Schmidt et al., 2015; Hafidh et al., 2016; Johnson et al., 2019). After the formation of male and female gametophytes, double fertilization is achieved when one sperm cell fuses with the egg cell, forming the zygote. Simultaneously, the other sperm cell fuses with the diploid central cell to form the endosperm (Faure, 2001). It serves to nourish the developing embryo until the onset of functional photosynthesis by the developing seedling, which represents the next generation. Moreover, the endosperm is economically very important, as it constitutes the seed component of cereal grains that supply the food industry worldwide (Schmidt et al., 2015; Skinner and Sundaresan, 2018; Johnson et al., 2019; Adhikari et al., 2020).

Considering that the formation of the two main outputs of fertilization (zygote and endosperm) greatly affect seed vigor, several key mechanisms coordinate the multiple stages of sexual reproduction in Angiosperms to ensure the correct development of gametophytes (Sprunck, 2020). Among the different regulatory mechanisms controlling sexual reproduction in plants, the post-translational regulation via ubiquitin proteasome pathway/system (UPP/UPS) has gained special attention in the past years, because it seems to play an important role in the process (Bolaños-Villegas et al., 2018).

The UPP/UPS is an irreversible process that targets specific proteins with ubiquitin moieties (Ub) for degradation in a spatiotemporal manner, controlling the abundance/activity of proteins required for a multitude of developmental processes. Nevertheless, after the reaction has been concluded, the free and reusable ubiquitin is released (Vierstra, 2009; Ciechanover and Stanhill, 2014).

The ubiquitination reaction consists in a combined action of three enzymes: ubiquitin-activating enzyme (E1); ubiquitin-conjugating enzyme (E2); and ubiquitin ligase enzyme (E3). First, in an ATP-dependent manner, the E1-activating enzyme forms a thioester bond with the C terminus of Ub and transfers the activated Ub to an E2-conjugating enzyme, and then the E3 ubiquitin ligase enzyme transfers the Ub molecule onto a lysine residue of the substrate (Peters, 2006). This labeling process is repeated several times, forming a chain of ubiquitin on the substrate, leading to their recognition by the 26S proteasome, a very large multicatalytic protease complex that degrades ubiquitinated proteins into a small peptide (Smalle and Vierstra, 2004).

The specificity of ubiquitin-dependent proteolysis is achieved at the level of substrate ubiquitination, which provides the E3 ligase enzymes with key roles in several cellular processes, especially in the cell cycle (Mazzucotelli et al., 2006; Teixeira and Reed, 2013; Shu and Yang, 2017). In this review, we will focus on the role of a particular class of E3s ubiquitin ligase enzyme in the model plant *Arabidopsis thaliana*, called Anaphase-Promoting Complex/Cyclosome (APC/C). Although its involvement in the cell cycle is widely understood, little is known about its function during sexual reproduction in plants.

## The APC/C E3 Ubiquitin Ligase Complex

As an essential E3 ubiquitin protein ligase involved in cell cycle regulation, APC/C is well known to mediate mitotic transition and mitosis exit by targeting essential cell cycle proteins, such as mitotic cyclins and securins, for destruction by 26S proteasome (Sudakin et al., 1995; Clarke et al., 2005).

Although it is understood that APC/C function is not limited to cell cycle regulation, only in the past years has an increasing number of works that report its role in plant development, such as cellular differentiation (Blilou et al., 2002; Lin et al., 2020), vascular development (Marrocco et al., 2009), shoot branching (Lin et al., 2012, 2020; Xu et al., 2012), root growth (Lin et al., 2020), hormone signaling (Blilou et al., 2002; Lin et al., 2020), epigenetic regulation (Zhong et al., 2019), male and female gametophyte development (Capron et al., 2003b; Kwee and Sundaresan, 2003; Zheng et al., 2011; Wang et al., 2012), and embryogenesis (Wang et al., 2012, 2013; Guo et al., 2016).

The APC/C is a large molecular machine highly conserved among eukaryotes, which has been proven by the ability of its genes from different species to complement the corresponding yeast mutant (Capron et al., 2003a; Eloy et al., 2011; Heyman and De Veylder, 2012; Wang et al., 2012). It contains several subunits, and in Arabidopsis at least 14 core subunits were identified: APC1, APC2, APC3a, APC3b, APC4, APC5, APC6, APC7, APC8, APC10, APC11, APC13, APC15, and CDC26 (Uzunova et al., 2012; Eloy et al., 2015; Lorenzo-Orts et al., 2019), each of them encoded by a single gene, except for APC3, which is encoded by two genes, APC3a/CDC27a and APC3b/CDC27b/HOBBIT (Pérez-Pérez et al., 2008).

The plant APC/C, based on homology with other organisms, holds different functional/structural modules (Eloy et al., 2015; Alfieri et al., 2017). The catalytic module comprises APC2, the CULLIN subunit, and APC11, the RING-H2 subunit, which are sufficient to catalyze *in vitro* ubiquitination reaction without substrate specificity (Tang et al., 2001). The APC10, known as DOC1 subunit, together with CELL DIVISION CYCLE 20 (CDC20) and CELL CYCLE SWITCH 52 (CCS52/CDH1), is responsible for substrate recognition, giving specificity for the reaction (Passmore et al., 2003; Da Fonseca et al., 2011). The APC3a, APC3b, APC6, APC7, and APC8 subunits contain tetratricopeptide repeat (TPR) domains, which are important for protein-protein interactions and assembling of the structural module (D’Andrea and Regan, 2003; Alfieri et al., 2017). APC13 and APC15 function as TPR-accessory subunit, which interacts with TPR subunits (Zachariae et al., 1998; Schwickart et al., 2004; Thornton et al., 2006; Chang et al., 2014, 2015; Alfieri et al., 2017). Although, already identified in the Arabidopsis genome, APC15 has not been characterized in plants, missing information about its function (Uzunova et al., 2012). The largest subunit is APC1, which contains another type of motif named proteasome-cyclosome (PC) repeat, important for APC/C stability (Lupas et al., 1997; Chang et al., 2014, 2015; Alfieri et al., 2017). APC1, together with APC4 and APC5, subunits, constitutes the platform module of the complex, to which the catalytic and structural components are attached (Thornton and Toczyski, 2003; Thornton et al., 2006; Schreiber et al., 2011; Chang et al., 2014, 2015; Eloy et al., 2015; Alfieri et al., 2017). More recently, the AtCDC26 has been identified as a distinct bicistronic transcript showing an upstream open reading frame (uORF) encoding a functional protein, which is part of the APC/C (Lorenzo-Orts et al., 2019). The *CDC26* subunit shows a monocistronic gene architecture in most eukaryotes species, however in the plant kingdom it is encoded by a bicistronic transcript upstream of the *TRIPHOSPHATE TUNNEL METALLOENZYME 3* (*TTM3*). Functional analysis of CDC26 showed that its ubiquitous expression completely rescued *ttm3-2* embryo lethality and *ttm3-1* defective root and hypocotyl growth, suggesting that the observed phenotype in these mutants is due to the lack of CDC26 rather than TTM3. Moreover, *ttm3-1* mutant plants crossed with CYCB1;1-GFP marker line showed reduced expression of GFP-expressing cells, indicating that the mutant is impaired in cell division and CYCB1;1-GFP protein stability (Lorenzo-Orts et al., 2019).

In order to perform its function, APC/C machinery needs a tight spatiotemporal regulation, meaning that different proteins interact with it to activate or inactivate its function. It is already well described that the catalytic activity and substrate specificity are conferred by two structurally related co-activators proteins, CDC20 and CCS52. Both proteins belong to a class of WD-40 repeat proteins, characterized by the presence of tandem repeats named after a high frequency of tryptophan (W) and aspartic acid (D) pairs, which should form a β-propeller structure and represent the major site for protein interactions (van Leuken et al., 2008; Eloy et al., 2015).

In Arabidopsis, five different genes encode putative *CDC20* (*CDC20.1*–*CDC20.5*). CDC20.1 and CDC20.2 were shown to be functionally redundant in mitosis, but not in meiosis (Kevei et al., 2011; Niu et al., 2015). However, the three other genes (*CDC20.3*, *CDC20.4*, and *CDC20.5*) appear to be pseudogenes that have lost their function as canonical *CDC20* genes (Kevei et al., 2011). Concerning *CCS52*, Arabidopsis contain two A types (*CCS52A1* and *CCS52A2*) and one B type, which is plant-specific (*CCS52B*) (Tarayre et al., 2004; Fülöp et al., 2005).

Among the identified APC/C inhibitors described in plants, there is the ULTRAVIOLET-B-INSENSITIVE 4 (UVI4) that regulates APC/C activity by binding to CCS52A1 co-activator. The UVI4 is a specific inhibitor of APC/C^*CCS*52*A*1^, and *uvi4* Arabidopsis mutants exhibit a smaller root meristem size, as a consequence of a reduced number of meristematic cortex cells, most likely due to an increase in APC/C^*CCS*52*A*1^ activity (Van Leene et al., 2010; Heyman et al., 2011, 2017; Iwata et al., 2011). Likewise, OMISSION OF SECOND DIVISION 1 (OSD1)/GIGAS CELL1 (GIG1), an UVI4 homolog (UVI4-like), was also found to negatively regulate APC/C activity by interacting with a range of APC/C activators, which include: CDC20.1, CDC20.5, CCS52A1, CCS52A2, and CCS52B. Moreover, it has been shown that double mutation of *OSD1/GIG1* and *UVI4* is lethal, suggesting that these genes are at least partially redundant. However, it is also noticed that there may be some functional differences between OSD1/GIG1 and UVI4 because their loss of function causes different phenotypes which are affected differentially by the overexpression of *CCS52B* and *CDC20.1* co-activators (Van Leene et al., 2010; Iwata et al., 2011; Cromer et al., 2012). Another plant-specific regulator that directly interacts with APC/C is SAMBA, found by tandem affinity purification through a direct interaction with APC/C. *Samba* knockout plants display enlarged meristems size, and produces larger seeds, leaves and roots, demonstrating that the gene plays a key role in organ size control. Moreover, biochemical analyses showed that the lack of SAMBA stabilizes CYCA2;3, pin pointing it as a plant-specific regulator of APC/C involved in the degradation of A type cyclins (Eloy et al., 2012).

## The Role of APC/C During Meiotic Cell Division

Progression from meiosis I to meiosis II requires a fine-tuned regulation of Cyclin-CDK activity, which must be partially reduced to exit meiosis I, but not completely abolished. It needs to be sufficiently high to allow the cell to directly enter the second meiotic division without replication, preventing its exit from meiosis at the first division (Marston and Amon, 2004; Pesin and Orr-Weaver, 2008; Mercier et al., 2015). The meiotic process relies on many of the same cell cycle regulators that act in mitosis. However, although not completely understood, the APC/C activity should be differentially regulated in meiosis in order to ensure proper segregation of homologous chromosome during meiosis I and sister chromatid separation during meiosis II (Pesin and Orr-Weaver, 2008; Wijnker and Schnittger, 2013).

As aforementioned, proteolytic regulation of Cyclins via APC/C is a critical step for cell cycle progression, once they enable the phosphorylation activities of CDK, allowing the cycle transition. During meiosis, there are two cyclins shown to have a meiotic function, the A-type Cyclin, CYCA1;2, known as TARDY ASYNCHRONOUS MEIOSIS (TAM), and SOLO DANCERS (SDS), a Cyclin with properties of both A- and B-type cyclins. CYCA1;2/TAM was demonstrated to be important for the transition between meiosis I and II (Figure 2). The complete suppression of *CYCA1;2/TAM* in Arabidopsis produces diploid gametes instead of haploids as a consequence of premature exit from the meiotic cycle, after the first division (Prophase I stage) (Magnard et al., 2001; Wang et al., 2004; Bulankova et al., 2010; d’Erfurth et al., 2010). Furthermore, plants that have a non-degradable version of CYCA1;2/TAM provokes the entry into an aberrant third meiotic division (Cromer et al., 2012). Regarding SDS Cyclin, it was shown that *sds* mutant failed in pairing homologous chromosomes, being unable to stablish crossing-over during prophase I, which results in reduced levels of meiotic recombination (Figure 2; Azumi et al., 2002; De Muyt et al., 2009). However, it has not been proven whether SDS is regulated via APC/C in a proteolytic fashion.

B-type cyclins are well known for having notable function during mitosis, and presently CYCB3;1 is the only B-type cyclin identified during meiosis via promoter:GUS reporter lines (Bulankova et al., 2013). Two *CYCB3;1* mutant alleles, *cycb3;1-1* and *cycb3;1-2*, generate pollen mother cells (PMCs) with uncommon structures, similar to an incomplete cell wall formation at ectopic locations. These cellular observations point to the CYCB3;1 function, which warrants the accuracy of cell wall formation in PMCs (Bulankova et al., 2013). Furthermore, reinforcing the role of CYCB3;1 in meiosis, the CYCB3;1-GFP reporter line was found to accumulate in the cytoplasm of meiocytes through prophase I, seeming to be associated with the spindle at metaphase I, however it was not present in metaphase II, possibly due to a proteolytic degradation during this phase (Figure 2; Sofroni et al., 2020).

Another important gene involved in the transition from meiosis I to meiosis II is *OSD1/GIG1* (Figure 2), which is known to inhibit APC/C activity and accordingly to promote CDK activity. *osd1* mutants exhibit reduced CDK activity, which avoids entry into meiosis II, and as a consequence diploid gametes are formed, similar to the phenotype observed in *tam* mutants (d’Erfurth et al., 2009, 2010; Cromer et al., 2012). However, when *tam*/*osd1* double mutant was analyzed, the results showed that it was male sterile, but female fertile. The *tam/osd1* double mutant exhibits the same female meiosis phenotype as the single mutants (*tam* or *osd1*), but male meiocytes apparently generate spores after prophase I without chromosome segregation. Therefore, these results reveal that there is a prominent difference in the control of male and female meiotic cell cycle progression that requires further investigation to determine precisely how it works (d’Erfurth et al., 2010).

The most recent player that has been shown prominent function during male meiosis is the *APC8* subunit. Meiocytes from *apc8-1* plants exhibit several meiotic defects including improper alignment of bivalents at metaphase I, leading to the production of dysfunctional tetrads with four nuclei containing numerous amounts of DNA. Showing that APC8 plays a role in meiotic chromosome segregation at metaphase I. Moreover, at metaphase II, *apc8-1* chromosomes are misaligned providing an unequal chromosome segregation of sister chromatids at anaphase II, giving additional evidence that APC8 is required for chromosome segregation in meiosis II (Xu et al., 2019).

The APC/C co-activator, CDC20.1, is also reported to be essential for normal male fertility and meiosis division. Disruption of *CDC20.1* results in incomplete alignment of bivalents at metaphase I, leading to unequal chromosome segregation in both meiosis I and II (Figure 2; Niu et al., 2015).

The THREE DIVISION MUTANT1/MALE STERILE 5 (TDM1/MS5) is proposed to be a putative meiotic APC/C component, and in agreement with this hypothesis, it displays structural similarities with the TPR domains of the APC/C subunits, in addition to interacting with APC3b and with the co-activator CDC20.1 (Cifuentes et al., 2016). TDM1/MS5 ensures that meiotic termination occur after the end of meiosis II (Figure 2). Mutation in TDM1/MS5 leads to an abnormal third division, similar to what is observed through the expression of a non-degradable version of CYCA1;2/TAM (Ross et al., 1997; Glover et al., 1998; Bulankova et al., 2010; Cromer et al., 2012; Cifuentes et al., 2016). Furthermore, TDM1/MS5 is inhibited at meiosis I through phosphorylation by CDKA;1-CYCA1;2/TAM complex, which prevents premature meiotic exit. In meiosis II, TDM1/MS5 promotes meiotic termination by activating the APC/C and/or by modifying its specificity (Cifuentes et al., 2016).

The SUPPRESSOR with MORPHOGENETIC EFFECTS on GENITALIA 7 (SMG7), which belongs to a family of plant-specific proteins, is partially similar to Xe-p9, a regulatory subunit of the Xenopus CDK (Glover et al., 1998; Bulankova et al., 2010). SMG7 is described to have a conserved role in nonsense-mediated RNA decay (NMD) in yeast and animals, and it is also reported be essential for the progression from anaphase to telophase in the second meiotic division in Arabidopsis, therefore being required for exit from meiosis (Figure 2; Riehs et al., 2008). *Smg7* mutants are arrested at anaphase II, likely caused by a failure to downregulate CDK activity after chromosome segregation in meiosis II, which requires TDM1 function (Riehs et al., 2008; Bulankova et al., 2010; Riehs-Kearnan et al., 2012; Mercier et al., 2015; Cifuentes et al., 2016).

Recent studies have demonstrated that SMG7 and TDM1 act in the same pathway to promote exit from meiosis. The phenotypes related to *smg7* and *tdm1* are consistent with the theory that both genes work together at the end of meiosis, downregulating CDK activity and promoting G1 phase transition. Epistatic analysis shows that SMG7 acts through TDM1, but it is currently unknown whether TDM1 is a direct target of SMG7 regulation (Bulankova et al., 2010; Cromer et al., 2012; Cifuentes et al., 2016).

Among the substrates, PATRONUS 1 and PATRONUS 2 (PANS1 and PANS2), have been characterized as an APC/C substrate. The PANS1, also known as COPPER MODIFIED RESISTANCE1 (CMR1), was previously identified by its reduced fertility due to low pollen viability and a female gametophyte developmental arrest, caused by a premature loss of sister chromatid cohesion before metaphase II (Cromer et al., 2013). The same gene was also named as CMR1, due to the identification of a mutant Cu^2+^ sensitive, caused by EMS mutagenesis. The results showed that PANS1/CMR1 was not only required for Cu tolerance but also more generally for survival under various environmental stresses, in particular salt stress, playing a key role in growth adaptation to stress (Juraniec et al., 2014, 2016).

More recently, PANS1/CMR1 has been elegantly shown as the Arabidopsis homolog of Securin (Cromer et al., 2019). Securin is an APC/C substrate in animals and fungi, involved in the control of the metaphase to anaphase transition and anaphase onset by inactivating the cohesion-cleaving enzyme, Separase (Marangos and Carroll, 2008).

PANS1 is a key inhibitor of SEPARASE, the enzyme responsible for triggering anaphase by cleaving Cohesin, which is the complex holding the sister chromatids together. Separase must be tightly regulated to prevent the precocious release of chromatid cohesion and a catastrophic chromosome separation. At the onset of Anaphase, the APC/C triggers the degradation of PANS1, releasing SEPARASE activity and allowing chromosome segregation (Cromer et al., 2019).

Furthermore, during meiosis depletion of PANS1 leads to the premature release of cohesion (Cromer et al., 2013). Expression of an APC/C-insensitive PANS1 abolishes cohesion release and chromatid separation, mimicking the depletion of SEPARASE (Liu and Makaroff, 2006; Cromer et al., 2019). Disruption of *PANS1* is known to lead to the premature separation of chromosomes in meiosis, and the simultaneous depletion of *PANS1* and *PANS2* is lethal, showing to be essential for sister chromatid separation during meiosis. It was observed that in *pans1* mutant, sister chromatid cohesion is lost before metaphase II, causing defects in chromosome segregation at meiosis II (Figure 2) (Cromer et al., 2019).

## Activity of APC/C Subunits at the Post-Meiotic Stage of Gametophyte Development

**FIGURE 3:**
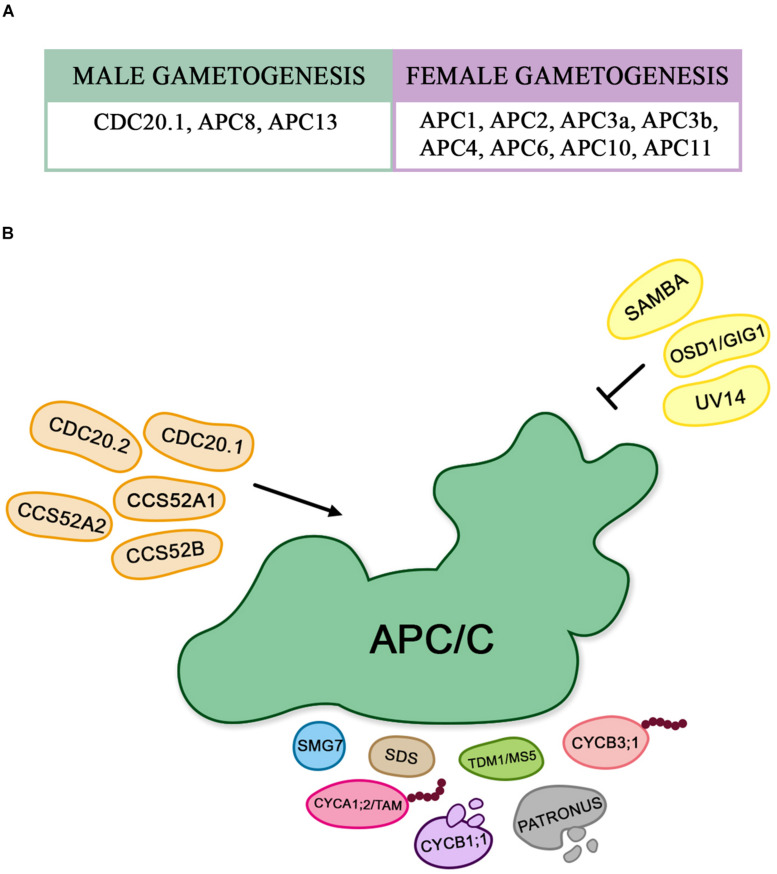
**(A)** The described APC/C subunits associated to female and male gametogenesis control. **(B)** Proteins that interact with the APC/C during gametogenesis: The schematic representation shows the different substrates of APC/C, and the proteins that interact with it by activating and/or inhibiting its function during gametophytic development in Arabidopsis.

Subsequently the meiotic process, the formation of male and female gametes has also been reported to be regulated by APC/C. In Arabidopsis, functional characterization of all APC/C subunits has been shown to be essential for gametophytic development, and consequently important to succeed in the double fertilization process (Figure 3A).

Mutations in the genes encoding APC/C subunits in Arabidopsis have been described as essential for gametophytic development and/or embryogenesis. Deletions in one of the alleles of *APC2* or *APC6* revealed arrested female gametogenesis due to a failure in degrading CYCB1;1, as shown through CYCB1;1 accumulation in megagametophyte (Capron et al., 2003b; Kwee and Sundaresan, 2003).

Single *apc10* and double *apc3a/apc3b* mutants show that APC10 and APC3 are also indispensable for female gametophyte development, although no mechanism has been investigated to show where the genes might play their role (Pérez-Pérez et al., 2008; Eloy et al., 2011). Moreover, APC1, APC4 and APC11 are also essential for female gametophytic development, and critical for embryogenesis too (Wang et al., 2012, 2013; Guo et al., 2016).

Genetic and cell biological analyses of the *apc11* mutant showed a zygote-lethal phenotype. Lack of *APC11* hampered the first division of the zygote, and also the over-accumulation of CYCB1;1D-box-GUS in arrested ovules (Guo et al., 2016). *Apc1* and *apc4* mutants accumulate CYCB1-GUS in ovules and seeds, showing their inability of CYCB1 degradation (Wang et al., 2012). The female gametogenesis of *apc1* and *apc4* was disrupted, leading to anomalous nuclei, as well as nuclear number and positions at variable stages, whereas embryogenesis was arrested at all developmental stages (Wang et al., 2012, 2013).

Contrastingly, disruption of *APC8* or *APC13* was shown to be involved in the male gametophyte development (Saze and Kakutani, 2007; Zheng et al., 2011). Disturbance on APC8 and APC13 function provoke failure of CYCB1;1 degradation (Zheng et al., 2011). Both *apc8* and *apc13* mutants are affected in pollen development, giving rise to an increased proportion of uni-nucleated mature pollen. CYCB1;1-GFP is accumulated in vegetative nuclei and expanded expression in sperm cell nuclei at the tricellular stage is observed in both mutants, suggesting that APC/C is required for removal of CYCB1;1, playing essential role during mitotic cell cycle progression during male gametophyte development (Zheng et al., 2011).

Likewise, SAMBA is believed not to act during meiosis, since *samba* homozygous mutants did not have their meiotic progression affected, but underwent post-meiotic changes, where the loss of *SAMBA* expression specifically interfered with mitosis I, resulting in pollen without sperm nuclei. Moreover, SAMBA is expressed during embryogenesis, indicating a possible role of *SAMBA* during this process (Eloy et al., 2012).

In Figure 3B, we show an overview of the proteins that are associated with APC/C during gametophyte development.

## Conclusion

The basic processes controlled by ubiquitin-mediated proteolysis in plants are very similar to other eukaryotes, although concerning the APC/C machinery control in plants, it seems that the complex and its subunits individually have some unique characteristics that are beyond cell cycle control.

The role of APC/C in plant development, therefore in plant reproduction, is likely to be dependent on gene structure and expression, which is revealed by the unique characteristics of the plant APC/C. Such unique characteristics are demonstrated by the particular assembling of flexible (sub)complexes, which may be required for specific plant growth responses needed in order to adapt to changing environmental conditions.

It is likely that the functions performed by some subunits during plant reproductive developments are dependent on the importance of the modules to which they belong, meaning that the severity of the phenotype of APC/C subunit mutants is determined by the degree to which the APC/C is affected (Wang et al., 2012).

Nevertheless, further research is still necessary to fully understand the molecular mechanisms regulated by APC/C during gametophyte development in plants. Moreover, to shed light on the mitotic, post-mitotic and meiotic processes, identification of its specific targets is essential to conclude at which stages of plant development the complex is required.

Despite its importance, the topic is still little explored, which might be due to the limitations of available mutants, we believe that in the coming years, we will be able to improve the subject using new accessible technologies, such as CRISPR-Cas9, for producing knockdown or knockout mutants from different plant species, using them for further studies.

## Author Contributions

NBE conceived the manuscript. MLSS wrote and NBE revised and corrected the article. IAR prepared the figures. All authors contributed to the article and approved the submitted version.

## Conflict of Interest

The authors declare that the research was conducted in the absence of any commercial or financial relationships that could be construed as a potential conflict of interest.
